# On the Identification of Body Fluids and Tissues: A Crucial Link in the Investigation and Solution of Crime

**DOI:** 10.3390/genes12111728

**Published:** 2021-10-28

**Authors:** Titia Sijen, SallyAnn Harbison

**Affiliations:** 1 Division Human Biological Traces, Netherlands Forensic Institute, Laan van Ypenburg 6, 2497 GB The Hague, The Netherlands; 2 Swammerdam Institute for Life Sciences, University of Amsterdam, Science Park 904, 1098 XH Amsterdam, The Netherlands; 3 Institute of Environmental Science and Research Limited, Private Bag 92021, Auckland 1142, New Zealand; sallyann.harbison@esr.cri.nz; 4 Department of Statistics, University of Auckland, Private Bag 92019, Auckland 1142, New Zealand

**Keywords:** forensic, review, body fluid, organ, tissue, identification, mRNA, DNA methylation, activity level

## Abstract

Body fluid and body tissue identification are important in forensic science as they can provide key evidence in a criminal investigation and may assist the court in reaching conclusions. Establishing a link between identifying the fluid or tissue and the DNA profile adds further weight to this evidence. Many forensic laboratories retain techniques for the identification of biological fluids that have been widely used for some time. More recently, many different biomarkers and technologies have been proposed for identification of body fluids and tissues of forensic relevance some of which are now used in forensic casework. Here, we summarize the role of body fluid/ tissue identification in the evaluation of forensic evidence, describe how such evidence is detected at the crime scene and in the laboratory, elaborate different technologies available to do this, and reflect real life experiences. We explain how, by including this information, crucial links can be made to aid in the investigation and solution of crime.

## 1. Activity-Level Evaluations: The Context of Body Fluid and Tissue Identification

Body fluid and tissue identification can add evidence in criminal investigations by establishing a crucial link between the donor, the cell type and the activities that occurred. In this review, we first provide the forensic context of body fluid and tissue identification which resides in activity-level evaluations, then we discuss methods to locate body fluids at the scene or in the laboratory, which is followed by the major marker methodologies to analyze them. One of these methods is mRNA profiling, and we take a retrospective look at casework details and verdicts for mRNA cases in the Netherlands. This leads us to a criminalistic view on targets that can be accommodated in assays and the interpretation of cell typing results for instance, in mixed stains, which needs a very different approach from the interpretation of DNA results. Lastly, we consider possible other marker types that have been suggested, and practical aspects when introducing cell typing into a forensic laboratory.

### 1.1. Prosecution and Defense Scenario

**Figure 1:**
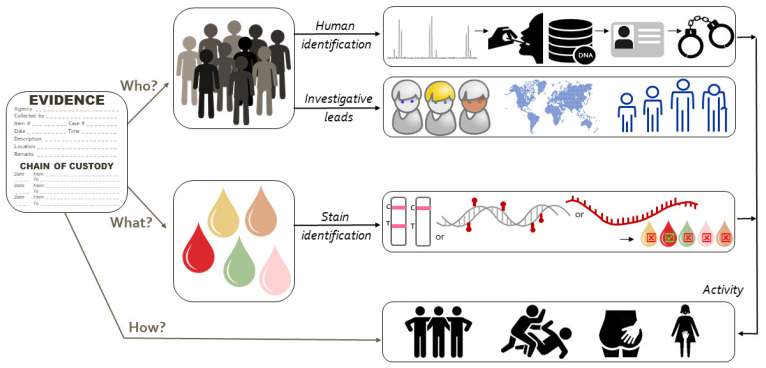
Stain identification in the context of the forensic process showing the link between ‘who’, ‘what’ and ‘how’.

Increasingly, cases come to court in which the presence of cellular material of a person is not disputed but the activity that caused the deposition is. The debate then centers around the question ‘how did his/her DNA get there’? Forensic scientists approach such questions by performing activity-level evaluations [1,2,3,4,5,6,7,8]. Figure 1 shows activity-level evaluation in the context of forensic human biology analyses. Generalizing, activity-level evaluations weigh the likelihood of the forensic evidence under one scenario (hypothesis 1; H1, also known as prosecution scenario) versus an alternative scenario (hypothesis 2; H2, also known as defense scenario). The two hypotheses need to be formulated well, describe the alleged activities and be mutually exclusive. In the evaluation, the probability of the findings given the propositions is considered (not the probability of the propositions themselves) [1].

The ‘findings’ in the case can relate to cellular material matching victim or suspect which under H1 is indicative of an offensive activity (e.g., blood matching the victim on the blade of a knife indicative of the victim being stabbed; cellular material matching the suspect on the handle of a knife supporting the proposition that the suspect carried out the stabbing). Under H2, this questioned cellular material on the evidentiary item may result from a number of scenarios: (1) the cellular material was deposited not during the offense but at an earlier or later time (the suspect did not stab but picked up the knife after the stabbing); (2) the cellular material was not deposited by direct contact but was the result of secondary (or tertiary or further) transfer (the suspect shook hands with the real perpetrator who then handled the knife in the stabbing); (3) the item on which the cellular material was deposited belongs to the suspect but was used in the offense by another person (the knife (owned by the suspect who uses it to cut food) was handled by the real perpetrator in the stabbing); (4) the cellular material was deposited on the knife in a different way (the victim fell into the knife).

### 1.2. Addressing Alternative Scenarios

Forensic studies aim to develop approaches to assist in addressing offensive and alternative scenarios. To address deposition during the offense or during an earlier or later encounter, inferring the time since deposition (TSD) may be helpful. For bloodstains, various spectroscopic and chemometric TSD methods have been proposed that rely foremost on changes to hemoglobin [9,10,11,12,13]. An approach that would be applicable to more body fluids than blood is based on differences in the degradation over time of distinct RNA molecules outside the human body. Determining relative expression ratios may allow inference of TSD, although robustness is not yet at the level needed for forensic casework [14,15,16,17,18,19]. Recently, microbes were explored for TSD purposes of saliva samples with some success, but the high inter-individual variation can surpass the time-dependent variation [20].

To address the question of primary deposition or secondary transfer, TPPR (transfer, prevalence, persistence and recovery) issues are studied [21,22,23,24,25,26,27,28,29,30,31]. The aspect of transfer appears the most complex among TPPR issues and many studies have gained information on transfer rates, and the various aspects that influence transfer rates such as wet or dried state, application of friction and pressure, smooth or rough surface and size of the stain [32,33,34,35,36,37,38]. Generally, probabilistic frameworks are used to guide the evaluation, and Bayesian networks are appreciated for their graphical strength in this evaluative process [39,40].

Inferring the body fluid(s) present in stains is, in the context of secondary (or more) transfer, important for two reasons: (1) to assess the cellular content of the primary stain in relation to the DNA recovered from the evidentiary sample as some fluids have very high cellular content (for example nasal secretion), whilst others contain much fewer cells (for instance urine) and (2) to assess whether the body fluid involved is likely to occur in what is suggested) to be the primary location [41]. For example, intimate body fluids (vaginal cellular material, menstrual secretion, semen) can be found in underpants of the wearer, but are less expected on hands or touched items [42,43,44]. Saliva and nasal secretion, on the other hand, can reside on hands or items, and blood could occur when people have (small) wounds. Organ tissues (for instance, CNS (central nervous system), heart, or adipose tissue), on the other hand, are not expected on hands or on items.

The scenario that another person and not the suspect utilized an item (belonging to the suspect) on which cellular material was deposited in the offense, can relate to objects (e.g., a knife used for stabbing or a bottle that was inserted vaginally) or clothing/ shoes that appear to have been worn during the offense for instance because blood spatter or organ tissue matching the victim found on them. Cell typing of samples from such items may provide the crucial link of the evidentiary item to the alleged crime (the knife carries muscle tissue matching the victim on the blade; the bottle contains vaginal material matching the victim on the bottle neck; the shoe carries CNS tissue matching the head-kicked victim), but the link to the perpetrator needs to be provided by other means. This may be addressed for instance through fingerprint analysis (e.g., on the bottom of the bottle or the handle of the knife), DNA-analysis (e.g., of the shoelaces or the insides of the neck and collar areas of the sweater) or CCTV-footage or witness reports on the suspect acting in the stabbing or wearing the shoes or clothing at the time of the attack.

When a different way for the deposition of the cellular material is proposed (e.g., shooting out of self-defense, intercourse with consent), the evaluation is often at the offense level, which means the domain of the court [2,45,46]. With such scenarios, DNA nor cell typing add value in most of the cases.

### 1.3. Contextualizing Evidentiary Traces

Not only the type of body fluid or organ tissue, but also the location, pattern and amount of cellular material can be relevant for activity-level evaluations or contextualizing the scene. For example, trousers of a suspect may carry multiple bloodstains matching the DNA profile of a deceased person. Bloodstain pattern analysis may reveal a mist-like pattern and diluted bloodstains. RNA analysis of multiple stains could indicate the presence of blood, saliva, and nasal mucosa in various combinations. Together, the location, pattern, amount, and type of detected body fluids indicate expirated blood. Pathology may reveal injuries to the lungs or airways, which could have triggered the victim to expel blood mixed with air from the lungs through the nose or mouth. The combination of identified body fluids, location/ pattern and amounts would support the suspect being in the vicinity of the dying victim expelling blood and would provide less support for a scenario in which the suspect would visit the scene of the crime a day later.

## 2. Locating Biological Forensic Evidence

Locating and identifying body fluids and tissues is important for two main reasons: (1) to find traces related to the alleged crime which can then be collected and from which the identity of the perpetrator may be revealed by further testing; (2) to contextualize the traces as explained in Section 1.3 to provide information about what activities may have taken place. This can be done at the scene, where time is limited, using techniques that are mostly presumptive and visual in nature, or in a forensic laboratory (using samples and items collected at the scene) where more detailed analysis is possible.

### 2.1. At the Scene of the Crime

Here we describe technologies for locating latent biological evidence for use at the scene that can also be used in the laboratory. These include chemiluminescent sprays such as those based on luminol, safer and non-invasive alternative light sources and spectroscopic methods using handheld instruments. Handheld devices, and technologies that are non-destructive to the sample offer the greatest potential as these can be used to rapidly direct investigations and the collection of evidence.

#### 2.1.1. Chemical Sprays for Blood and Semen

Sometimes blood at crime scenes is difficult to see, perhaps there is a very small quantity of diluted blood (cleaning may have taken place), the blood is on a surface that makes it difficult to find (like a dark substrate or upon repainting) or there is a trail of blood-stained shoeprints leading from the scene which are no longer visible. Luminol and the more user-friendly, luminol-derived Bluestar (Bluestar Forensic, Monaco) reagent [47], are often used to detect haemoglobin and its derivatives resulting in a blue fluorescence which is captured by photography [48]. One disadvantage of such methods includes that the luminescence produced can be faint and short-lived, and in the case of luminol the visualization must be carried out in near darkness [48]. Different formulations exist and those that maximise the brightness and longevity of the fluorescence and that have few adverse effects on subsequent tests for blood or on DNA profiling are preferred [48,49,50,51]. A more recent development is the proposed use of a click reaction [52] between serum albumin and tetraphenylethene maleimide. The reaction is less harsh than that of luminol and BlueStar and produces a stable and sensitive response and shows promise for further development.

To locate semen, alternative light sources (ALS; see Section 2.1.2) and acid phosphatase tests are used that suffer from limitations in sensitivity and toxicity, respectively. In 2017, STK Sperm Tracker™ was introduced to the market by AXO Science (Lyon, France) as a human semen-specific test [53]. Sperm Tracker is available as a spray for crime scene use and in impregnated sheets for use on larger items. Detection requires a UV light source, that the room or space be darkened, and it must be used before luminol or BlueStar.

#### 2.1.2. Alternative Light Sources

ALS refers to the use of light of different wavelengths, including infrared, to detect forensic evidence in a non-destructive manner, and are often used to assist in the reconstruction of events. The detection of biological evidence is possible because they contain autofluorescent components which allow them to be visualized when illuminated at specific wavelengths.

An early study comparing different laser and ultraviolet light sources for the detection of biological fluids [54] has been followed by the development of many commercially available light sources such as the Polilight^®^ series (Rofin Australia Pty Ltd., Melbourne, VIC, Austraila) and the LED-based Crime-lite^®^ series (Foster and Freeman Ltd., Evesham, UK). A selection of illustrative articles is presented here.

In an examination of the utility of the Polilight^®^ [55] stains of blood, semen and saliva, not visible to the eye, were able to be detected and Polilight^®^ performed well when compared to other presumptive screening tests.

As different body fluids and substrates fluoresce at different wavelengths, selecting the best combination of lightsource (wavelength) and filter used for detection is important. By comparing results obtained from five body fluids applied to 29 different materials, the Lumatec Superlight 410 (Lumatec GMBH, Deisenhofen, Germany) was used on fresh stains [56] and the same stains after two years [57] using different filters. Their findings show that for every combination of substrate, fluid, light source, and filter, except one, there was a specific combination that was best. In general though, stains could still be visualised two years later, using a light source between 415 nm and 460 nm in combination with a yellow or orange filter.

To improve the utility of light sources and photography at crime scenes, a study was conducted [51] combining a blue Crime-lite XL light source in combination with a 360° camera to capture semen and saliva stains on a number of different substrates. This study was carried out in a mock crime scene indoor setting using volunteers to examine the images and locate the stains. The colour of the substrate was found to have an effect on the ability to see the stains, however, in general, the combination of light source and camera was effective.

A side by side comparison of the Crime-lite^®^ 82S, Polilight^®^ PL400 and Polilight^®^ PL500 [58] was recently carried out, comparing the sensitivity and performance of each on dilutions of saliva and semen on different fabrics. The LED light source used in the Crime-lite^®^ 82S provided better detection of dilutions of saliva and overall performed better as a screening tool.

Recent developments include combined handheld, UV/Vis/IR multi-wavelength light sources and camera combined, with automatic filter selection all controlled from an integrated touch screen for example the Crime-lite AUTO. This handheld instrument appears to offer a number of advantages for crime scene use, the most obvious of which is the combined handheld unit. See also Section 2.2.3 for an alternative.

#### 2.1.3. Spectroscopic Methods at the Scene

Spectroscopic techniques for locating and identifying body fluids are available, with handheld devices for crime scene use as well as methods requiring laboratory-based equipment [59]. Handheld NIR (near infrared) spectrometers have been tested [60] with blood and potential applications to other fluids including age estimation [13]. NIR spectroscopy is quick and non-destructive with no sample preparation and background fluorescence encountered with [61]. In the most recent example [60], the instrument is paired with a mobile phone and software containing the necessary tools to train a variety of statistical models to interpret test data based on machine learning. The best of these models could correctly identify blood in 94% of cases, albeit with a false positive rate of 14% for blood-like substances such as red wine, tomato sauce, and coffee.

Hyperspectral imaging (HSI) is also a non-destructive spectroscopic technique and can be used to “map” large geographical areas as well as smaller items [62]. HSI records, as a continuous measurement, electromagnetic spectra over a wide range of wavelengths for every pixel in an image, and from this creates unique spectra. Since 2012 when first proposed as a tool to characterise forensic evidence [63,64] HIS has emerged as a potential tool for bloodstain detection.

The identification of blood by HIS is based upon the visualisation of haemoglobin and its components in the 400 to 700 nm range. Changes in the Soret peak observed at 415 nm are used to detect aging of the sample. In a set of fingerprints, aged over a 30 day period, HSI enabled the identification of the blood as well as aging of the prints [65].

When comparing a Crime-lite^®^ ML2 ALS instrument (Foster and Freeman Ltd., Evesham, UK) and HIS-NIR imaging using an SWIR3 hyperspectral camera working in the 1000–2500 nm spectral range (Specim Ltd., Oulu, Finland) [66], the resultant images obtained from the HIS-NIR imaging were shown to be superior at revealing the location of body fluid staining in known reference samples, simulated casework samples and on unknown staining on forensic exhibits. Using blood samples and different substrates, spectra were generated [67] that were used to train different classifiers to detect aged bloodstains and most recently a “blood detection dataset” was produced [68] specifically to assist in the challenging task of algorithm development for this new technology.

### 2.2. At the Forensic Laboratory

In the previous section, we described approaches for locating biological evidence at crime scenes, particularly latent evidence that may not be visible to the naked eye. These methods can also be employed in a forensic laboratory when transportable items are involved. In the remainder of this section, we discuss methods that are primarily laboratory-based for locating biological fluids. These chemical, immunological, and spectroscopic tests are often used to presumptively identify biological fluids before conducting further, more specific testing where necessary.

#### 2.2.1. Chemical and Immunochromatographic Tests

With the exception of microscopic identification of spermatozoa, forensic scientists often rely on sensitive but non-specific chemical tests, based primarily on the activity of enzymes in the target body fluid. Although easy to use, they are not human-specific and are largely presumptive in nature with many examples of false positive reactions reported. For reviews covering this area, see [69,70,71].

To supplement these chemical tests, laboratories may use immunochromatographic assays, which include tests for saliva, semen, urine, blood and menstrual blood. Despite advantages of speed, cost and ease of use, these assays are not as suitable for crime scene deployment as they require an extract of a sample to be made and are not an integral part of the DNA profiling process. To date, there is only one report of combining such tests into a format amenable to detecting body fluids in mixtures [72]. The more recent emergence of proteomics in the forensic literature (see Section 7.1.2) and the identification of new candidate markers [73] is likely to drive the development of immunologically-based assays based on multiplexing in a single assay that may be amenable for use at the crime scene.

Immunochromatographic methods can also be used to detect DNA amplicons and direct PCR of *Streptococcus sanguinis* and *Streptococcus salivarius* specific to saliva has been successfully integrated with an immunochromatographic strip detection method [74].

Improvements to the technology for detection have allowed for the development of a protein microarray sensor [75] for the detection of semen and vaginal cellular material. Antibodies to semenogelin-2 and anti-17 beta estradiol were immobilised on an array surface. To enhance the signal achieved and increase the sensitivity of the assay, a metal enhanced fluorescence (MEF) approach was taken. The sample containing body fluids was itself labelled with a fluorescent dye and after hybridisation and washing steps the fluorescence signals are detected.

#### 2.2.2. Spectroscopic Techniques

Raman spectroscopy, for which less sensitive handheld devices are available, relies upon the scattering of light by biological compounds. The outputs are complex spectra built up of the multiple components of each fluid. Statistical tools are available and needed to determine the unique spectroscopic profile of the molecular structure of each. Such profiles have been determined for a range of body fluids and tissues of forensic interest, such as blood [76], semen, saliva, vaginal fluid and sweat [77,78].

Early evaluations [76] considered factors of forensic interest such as the substrate, within-person variability and time since deposition in blood samples. Blood was able to be detected on luminescent fabrics by first removing the stained area from the substrate. Variability was observed as bloodstains aged and also between and within people. The changes in spectra as a bloodstain ages were further explored [79] over a period of 2 years with age predicted with an accuracy of approximately 70%. Statistical models were further improved [80] to accommodate environmental factors.

Fourier transform infrared (FT-IR) spectroscopy, routinely used in forensic chemistry, has also been evaluated for biological fluids with an important consideration that any environmental contamination is accounted for. Attenuated total reflectance (ATF) FT-IR spectroscopy [81,82] has been used to characterise a number of body fluids, accommodating the age of the stain and excluding unexpected non-target components. Similar studies have also been undertaken on semen [83,84], with methods developed to identify and avoid interference from a number of environmental factors; on dried urine [85] where the gender of the donor was able to be deciphered; and to distinguish between menstrual and peripheral blood [86]. The alternative modification, external reflection FT-IR spectroscopy, has also been proposed [59] to specifically identify body fluids associated with sexual assault. The effects of different fabric colours and commonly used household substances on identification were also assessed. These instruments are not handheld and, therefore, not compatible with examinations at the crime scene.

#### 2.2.3. A Practical Example

The utility of combining ALS detection with an immunochromatographic test for the presence of prostate-specific antigen (PSA, also known as gamma-seminoprotein, kallikrein-3 or P-30 antigen), on laundered clothing [87] followed by DNA profiling has been demonstrated. In this example, semen stains were located using a Forenscope Mobile Multispectral UV-VIS-IR Imaging System^®^ (Grimed Ltd., Voor, The Netherlands) which, like the Crime Lite AUTO described in Section 2.1.2, combines light source, filters, camera and software in one handheld instrument. Semen stains could be detected and confirmed using both the Forenscope system and the PSA test. High washing temperatures had the biggest impact on the ALS detection, polyester fabric had the biggest detrimental impact on the PSA test. DNA profiling however was successful on all semen samples regardless of washing temperature, detergent or fabric.

## 3. Major Types of Markers and Technologies Employed to Detect Them

### 3.1. RNA Markers

RNA is the link between the genome and protein content of each cell and its expression or its effect on expression of cell type-specific proteins has been widely adopted as a laboratory-based approach for body fluid identification [69,71,88] for forensic purposes. Alongside mRNA, small non-coding RNAs have generated significant interest [89] to date, which will be described in Section 7.1.1. RNA analysis has also been suggested for other forensic applications such as post mortem interval and age of a stain determination [17,19,90].

#### 3.1.1. Messenger RNA

There are many examples, over more than twenty years, of messenger RNA (mRNA) being proposed as a confirmatory test for body fluid and tissue identification with an early example being the identification of menstrual blood [91]. A selection of the numerous papers published are included here and will assist in directing the reader to further studies.

The stability of RNA in stains of forensic interest was described in 1999 [92] with a demonstration of the detection of skin epithelial cells in dried blood cells. This was followed by studies describing stability studies in vaginal swabs, blood, semen and saliva stains [93], stains up to 23 years old [94] and transcriptome studies showing the persistence of regions of RNA of interest in samples deliberately degraded [95,96,97].

To minimise sample loss during testing and optimise the efficiency of the laboratory process, it is important to recover DNA and RNA from the same sample [98,99,100]. A recent paper compared a number of different approaches [101] and found methods with small modifications to [98,99] remained the best. Equally important in minimising sample loss and optimising the laboratory process is the creation of multiplex assays each containing mRNA markers of known specificity [98,102,103,104,105,106,107] that can be selected from the literature and online databases [98,107] and whole genome expression analysis [108,109,110]. Once these factors are considered, then casework and casework applications follow [111,112,113,114,115].

Most of the published work depends upon the detection of mRNA markers by reverse transcription PCR (RT PCR) and capillary electrophoresis (CE) [92,102,103,106,116]. Real time PCR (qPCR) has also been used [116,117] but is somewhat limited by the number of markers that can be multiplexed together (restricted by the availability of fluorescent dyes) and the difficulty in identifying appropriate housekeeping genes, applicable to all included body fluids of interest. More recently, massively parallel sequencing (MPS) has been used to detect multiplexed markers [118,119,120] reflecting a shift towards this technology in forensic science [121]. The main advantages of MPS is the high multiplexing capacity, that amplicons can have overlapping (small) sizes and that nucleotide variation is detected. These first two features are of importance with forensic traces as they are often of minute amounts and suffer from degradation due to external factors. The forensic importance of analysing sequence variation in RNA amplicons will be discussed in Section 6.4. Other techniques for detecting markers are given in Section 3.1.3.

#### 3.1.2. Circular RNA

Circular RNAs (circRNAs) are highly expressed [122,123] and as they share sequence with the mRNA equivalent to which they are related can also be body fluid/ tissue-specific [124]. Because these molecules are closed circular structures, they are very stable and this makes them promising candidates for body fluid identification. In the first report [125] of their inclusion for forensic application, microarray expression profiles of circRNAs of venous blood, semen, saliva, vaginal cellular material and menstrual blood samples were produced. Although semen, saliva and venous blood could be distinguished from each other vaginal cellular material and menstrual blood could not.

A further study [126] combined mRNA markers with circRNA markers for the same genes (ALAS2 and MMP7), which were markers already used for body fluid identification [106,127,128], and demonstrated improvements in the sensitivity and stability of the assay compared to mRNA alone. This was also found in a similar study of circRNA markers in blood, menstrual blood, saliva, semen, urine and vaginal cellular material, using gene targets well known in the mRNA profiling community (for example HTN3 for saliva and CYP2B7P1 for vaginal material) [129]. Further investigation resulted in an 18 plex multiplex assay combining circRNAs, mRNAs and housekeeping genes, with good sensitivity and specificity and the potential to identify fluids in degraded samples due to the stability of the circRNA molecules [129].

#### 3.1.3. Alternative Techniques to Analyse RNA Markers

Many of the methods and much of the research described above use either RT PCR and CE, qPCR and/or increasingly RNA sequencing to detect the markers under study. These have been described in more detail in a recent review [88]. Some examples of newly proposed techniques follow.

Loop mediated isothermal amplification (LAMP) [130] can provide higher specificity than PCR because of the use of multiple primers and the higher temperatures at which amplification occurs. In 2015, the first reports of applying LAMP to mRNA body fluid identification [131] detected hemoglobin beta as a marker for blood and was followed by similar studies using a direct RT LAMP method to detect the expression of the statherin gene in saliva [132] The same markers and the TGM4 gene marker for semen were targeted in the design of an assay for the identification of blood, semen and saliva [133]. Extending the format to include vaginal cellular material and azoospermatic semen in a microtitre plate with a metal dye indicator and colorimetric detection, significantly improved the utility of the approach and may make it more suitable to laboratory requiring a higher throughput [134].

High resolution melt (HRM) curve analysis measures the melting temperature (Tm) and generates a distinct and characteristic melt curve for each amplicon in a sample. It has been proposed for a number of applications such as a detection method for SNPs [135], to detect sequence differences in the hypervariable regions of mtDNA [136], to characterise the oral microbiome of different people using 16SrRNA [137], as a technique to analyse methylation sensitive sites for body fluid determination [138] and as a method to identify mRNA markers in body fluids [139] in a multiplex format due to the high resolution of each distinct melt curve for each amplicon.

Since two publications some time ago [108,140], the NanoStringR nCounter system (nanoString, Seattle, WA, USA) [141] has received little forensic interest. This is somewhat surprising given the strengths of the platform that include a direct assay not involving PCR and measurement of gene expression of up to 800 mRNA candidates in a single multiplex reaction in a semi-automated fashion. These studies successfully tested 18 and 23 markers for body fluids, respectively. [108,140]

Similarly, HyBeacon probes [142], used for the detection of SNPs and STRs in areas such as clinical diagnostics and food authentication, have received some but not extensive interest. The ParaDNA Body Fluid ID test comprising RT PCR and melt curve detection was successfully evaluated for the detection of mRNA in body fluids of forensic interest [143] in combination with the now withdraw/ unavailable ParaDNA system (LGC Forensics Teddington, Middlesex, UK) [144] for STR detection.

### 3.2. Epigenetic Markers

Body fluid identification can also be accomplished at the DNA level by examining epigenetic modifications to specific sites on the genome, primarily methylation at the 5′ position of cytosine in a CpG dinucleotide. These sites are known as tissue-based differentially methylated regions (tDMRs) and have been specifically examined for body fluids of forensic interest such as blood, semen, saliva, skin, urine, and vaginal secretions.

Whilst the initial application of epigenetics in forensic science was the identification of body fluids and tissues, methylation analysis has also been proposed for age estimation and differentiation of twins [145,146,147]. A range of methods have been applied for identifying appropriate markers, detecting the epigenetic modifications, and assessing the results. A comprehensive review [146], describes many of these approaches in more detail.

#### 3.2.1. Marker Selection and Assay Design

With the increasing availability of freely available methylation data, researchers can assess many thousands of CpG sites simultaneously to find candidate markers. For example, the human methylation bead array system (Illumina) was used to screen over 450,000 CpG sites in samples of blood, saliva, and vaginal cellular material [148,149]. More recently, overexpressed genes in genome wide expression datasets were combined with heavily methylated gene body CpG islands (CGI) from methylation datasets [150]. Further *in silico* analysis of one of these datasets [149] was undertaken and four new markers able to discriminate blood (2 markers), vaginal cellular material (1 marker) and buccal cells (1 marker) were found [151].

Once identified, primers are designed and ideally markers are multiplexed enabling the simultaneous identification of different fluids/tissues. An example is a PCR multiplex combining CpG sites in the DACT1, USP49, PFN3, and PRMT2 genes to identify spermatozoa and differentiate between menstrual blood and vaginal cellular material from blood and saliva [148,152]. This multiplex has been extended to include amplicons for the 16S rRNA gene of bacteria specific oral and vaginal origin, *Veillonella atypica* and/or *Streptococcus salivarius*, and *Lactobacillus crispatus* and/or *Lactobacillus gasseri* respectively [153].

#### 3.2.2. Techniques to Analyse Epigenetic Modifications

Early research focused on the use of methylation-sensitive/ dependent restriction enzymes followed by PCR and CE [154]. The advantage of this approach was the ability to co-amplify STRs at a similar level of sensitivity, but drawbacks included incomplete restriction and template degradation, which can distort the methylation ratios that are critical to the interpretation of the results, which was particularly pronounced in low template samples. More recent assays [155], therefore, use amplicons with multiple restriction sites.

There are multiple methods to determine the relative methylation levels achieved, and they all start with bisulfite conversion of the DNA and amplification using primers designed to anneal to the converted DNA. Examples are qPCR with HRM [138,151] often confirmed with pyrosequencing [156], sequencing of cloned products [150] and changes in mobility of the PCR amplicons [157]. A multiplex methylation SNaPshot approach has been used to identify blood, saliva, semen and vaginal cellular material, and produced successful DNA methylation profiles in aged and mixed samples [148]. In a separate study it was successful in the identification of semen, saliva, venous blood and menstrual blood in body fluid mixtures and in crime scene stains [158]. Methylation state specificity was achieved using the single-base extension primers of the SNaPshot assay. Amplification refractory mutation system-PCR (ARMS-PCR), otherwise known as allele-based PCR [159], in combination with CE was used as an alternative method to test 22 possible markers for venous blood, saliva, semen, menstrual blood, and vaginal cellular material in multiplexes [160]. A random forest model was employed and performed well in predicting single source body fluids with high prediction accuracy (99.66%).

The multistep process of bisulfite conversion, PCR and detection by CE or sequencing is vulnerable to variation due to poor sample quality and quantity and especially degradation during the bisulfite conversion step. A new multiplex quantitative RT PCR method to measure the amount of genomic and bisulfite-converted DNA, and the degradation level of the conversion step as well as the conversion efficiency has been described [161]. Besides, natural variation in methylation status has been found between individuals, and some tissue-specific differentially methylated regions are susceptible to change due to environmental factors and age. For example, methylation of a CpG site in PRMT2 in blood samples was found to be an age-associated marker, whereas no significant difference based on age was observed for three spermatozoa-specific hypomethylated markers DACT1, USP49, and PRMT2 in men of different ages [149,152]. Others have demonstrated that within-person variation in methylation ratios was not observed, but variation was observed between people in an assay designed to identify vaginal cellular material [157].

### 3.3. Microbial Markers

Microbiomes are comprised of the bacterial, archaeal, viral and fungal microbial taxa communities present in/on a location of interest, such as the human body. Microbes have an advantage of high abundance and stability. Microbiomes can be considered as a whole (the human microbiome) or more specifically relating to a more defined location, for example the skin microbiome of humans where different bodily locations (moist armpit, sebaceous facial areas, dry forearm) can be dominated by different species [162]. A subset of markers and target organisms can be chosen to represent the fluid/ body site of choice [153,163] but it should be noted that different species may perform the same biological function (such as the production of lactic acid in the vagina) and may not function in all individuals [164,165]. Even human blood, previously considered to be sterile in a healthy person, may not be so [166].

More routine analysis of microbiomes has been made possible by developments in sequencing and related technology. Despite early promise, some challenges remain in adopting this technology for forensic science. In addition to variations between and within people [164,167,168,169,170] a key consideration is that microbial communities are strongly influenced by many external factors such as geography, time, season, health, diet, genetic factors, anti-inflammatory drugs and lifestyle [171,172,173,174,175,176], and even the presence of pets [177]. Consideration of these factors is necessary for any evaluation of microbiomes whether they “match” or not. Many of these factors relating to the human skin microbiome are neatly summarized in [178,179] and can be directly applied to all microbiome research and applications. In particular, the potential effects of the presence of contamination in the reagents and consumables used has been highlighted [180].

#### 3.3.1. Microbial Markers for Body Fluid Identification

Many possible applications of the analysis of microbiomes are described in a recent review [181]. These include the identification of people [182,183,184]; establishing links with personal possessions such as phones and shoes [185,186,187]; forensic botany (including environmental DNA), in which analysis of the phyllosphere and soil can provide links between people and crime scene locations [171,188,189,190]; and the identification of microflora specific to different body locations such as saliva [191].

Many studies have focused on specific body sites/ fluids for example the microbiomes of saliva [192,193], faeces [194], the vagina [164,195] with recent publications focusing on distinguishing between different sources of blood (menstrual, venous, fingerpick and nasal) [196,197] and semen [198]. *Lactobacillus* species have been incorporated into mRNA multiplexes and collaborative exercises [163,199] and in multiplexed methylation -sensitive restriction enzyme PCR assays [153]. Interpretation of the presence of such markers should proceed with care as microorganisms can be detected on non-target body locations, probably either from their high abundance and/or carry-over [104].

Unsurprisingly, vaginal samples and menstrual blood are unable to be distinguished and share metagenomic profiles [196,200], and in one study, samples taken from the penis the vaginal markers responded while no female DNA was detected [168].

In an extension from simply identifying bacteria representative of the oral cavity, it was [201] recognized that identifying them could be used to distinguish between expirated bloodstains (such as those forced by airflow out of the nose or mouth) and impact spatter, providing what could be an additional crucial piece of information for an investigation (see Section 1.3).

#### 3.3.2. Technologies to Analyze Microbial Markers

For the identification of microbial profiles specific to body locations, sequencing of the 16S rRNA gene is typically carried out, with the profiles found to be diagnostic of the area sampled. Saliva, skin, peripheral blood, menstrual blood, faeces and semen have all been tested in this way [169,200,202]. Whole genome shotgun sequencing has been suggested to outperform 16S rRNA analysis because of higher accuracy [203], but this comes with higher costs.

Not all assays are based on the 16S rRNA gene or on sequencing. For example microarrays targeting multiple genes across a range of known microorganisms have been described [164,168] and a qPCR assay [193] has been developed to detect three bacteria (*Streptococcus salivarius*, *Streptococcus sanguinis*, and *Neisseria subflava*) known to be present in the oral cavity. A similar microarray-based method has been described [194] for targeting the 16S rRNA, GroEL and 18S rRNA genes of a number of microorganisms present in faeces and other forensically relevant fluids.

## 4. Retrospective Analysis of RNA Typing in Forensic Casework

### 4.1. Requests and Results for RNA Typing in Casework

Forensic casework comes in great diversity, and questions can be put forward that cannot be addressed with existing methodologies. This feeds the development of new methods, like RNA typing assays which were especially triggered by the limitations for the tests for vaginal cellular material and menstrual secretion [72,204,205,206,207] and the difficulties to apply immunohistochemical staining for organ tissues to samplings from bullets where the tissue tends to reside in the grooves [208]. It is interesting to reflect, in a retrospective manner, on which forensic questions RNA typing assays are applied in actual forensic casework, and whether this corresponds to the questions that initially triggered the development of the assays.

RNA typing has been applied regularly at the Netherlands Forensic Institute since 2012. RNA was extracted in 452 cases and in 238 of these cases, RNA typing was performed (in the other 214 cases, RNA was extracted but RNA typing was not (yet) requested by police or prosecution). In 178 cases (75%) body fluid typing was performed and in 60 cases (25%) organ typing. On average, RNA is extracted for four samples per case. To study the context and details of cases in which RNA typing was performed, the details of 27 body fluid cases analyzed in 2020 were examined. All 27 cases were sexual assaults, of which 25 questioned the presence of vaginal cellular material (note that at the NFI presumptive tests are used to assess the presence of blood, saliva, and seminal fluid). The 26th case aimed to discern menstrual or peripheral blood in a vaginal sampling of an assaulted female and the 27th case questioned the presence of semen or seminal fluid on the genitals of an adolescent female (generally the presence of semen is not assessed through RNA typing but microscopic analysis and differential extraction is used). Thus, in the Netherlands, RNA body fluid typing is mostly applied in sexual assault cases to assess the presence of vaginal cellular material.

In the 25 cases that assessed vaginal cellular material, 56 samples were analyzed of which 19 resulted in vaginal cellular material detected (13 cases). In nine samples (four cases), saliva was detected (one of these samples, a penile swab, was also positive for vaginal cellular material; the other saliva-positive samples were all finger/nail dirt samplings). The corresponding DNA profiles of 53 of these 56 samples were mixed male/female profiles, while three showed no male (amelogenin Y) DNA and a full female profile (samplings from paper tissue, condom, or finger). The male/female ratio at the amelogenin locus in these mixed samples varied from 0.8 to 178, but this bore no relation to whether vaginal cellular material was seen or not. This is not unexpected as these are casework specimens, not ground truth data, and the female component may derive from other cells, for example skin cells, which are no longer included in the NFI body fluid RNA assay used in 2020.

### 4.2. RNA Typing Results in Verdicts

The ultimate value of RNA typing is best assessed by regarding verdicts after trial procedures (the proof is in the pudding). In the Dutch registry of verdicts [209], 21 cases can be found in which RNA typing was actively considered in the verdict by the judge (note that not all verdicts are taken up in the registry and that the judge decided on the use of the RNA results in the context of all evidence presented, in most cases without data on prevalence of such RNA results in alternative scenarios).

Ten cases were sexual assaults and 11 cases were not. For the sexual assaults, in nine cases the presence of vaginal cellular material was of importance; in one case menstrual secretion. These body fluids were found four times on a penis, three times on a finger/nail dirt, once in underpants of a male, once on the outside of a condom and once on an inserted item (in this case a piece of ginger; a cultural punishment by a father). In eight of these ten cases, the defense presented alternative scenarios: once the timing of deposition was questioned (“the forensic report does not state when the menstrual secretion was deposited on the penis”). In six cases, secondary transfer was suggested: Three times vaginal material was supposedly picked up from either an item carrying vaginal material, or from being nearby the victim’s vagina or from an unspecified source and then transferred to the penis. Vaginal cells on fingers of the suspect were suggested to originate from the victim touching her vagina and then shaking hands with the suspect, or from an unspecified activity. Vaginal cells in male underpants were suggested to originate from assisting the victim during vomiting after which her cellular material was transferred to his penis and underpants after a toilet visit (RNA typing was performed much later so the alternative scenario focused on explaining the female DNA). For a case with the presence of vaginal cells on the outside of a condom, the alternative scenario was that the father masturbated using a condom and that the results of vaginal cells and DNA-match with the adolescent daughter should be ignored as the mother was not included in the comparison (note that the DNA-profile fully matched the daughter).

These alternative scenarios follow three of the four general scenarios proposed in Section 1.1. These were: (1) ‘time of deposition’, (2) ‘secondary transfer’ and (3) ‘innocent explanation’; ‘another person performed the crime’ was not proposed. This makes sense as most of the evidentiary items involved body parts such as finger/nail dirt or penis.

In the remaining 11 verdicts considering RNA typing results, the evidentiary items included two bullets, two knives, a baseball bat, five pieces of clothing and a bed sheet. The cellular material found on these items matched the victim(s) in all 11 cases. Of the 11 cases, nine cases were analyzed for organ tissues and two for body fluids. Both multiplexes contain markers for blood, and in all 11 cases, blood was present in the stains analyzed. In four cases, CNS tissue was found as well (not necessarily in all evidentiary stains analyzed within a case; all four cases involved samplings taken from clothing). In five cases, skeletal muscle was detected (both cases that involved a knife, one of the cases involving a bullet and two cases involving clothing: for these latter two cases, CNS was observed in stains analyzed within the case as well). In one of the two cases analyzed for body fluids, besides blood, saliva was found in a mist-like pattern indicating expirated blood (this case was analyzed using a body fluid assay not yet carrying nasal mucosa markers).

The alternative scenarios put forward by the defense fall into three categories: once the time of deposition was questioned (this was for the case in which expirated blood was implied; the suspect was suggested to have visited the scene a day later when the victims had already died). Five times it was suggested that another person was involved (twice handling the knife, three times wearing the clothing that contained CNS material matching the victim). Five times another explanation was given (the suspect shot at a car not at the victim; the victim started shooting first so it was self-defense; the victim fell of the stairs (for the case in which blood was found on a baseball bat); the injuries were self-inflicted and the large puddles of blood on the bed sheets were staged by using animal or menstrual blood (this was the second case analyzed by body fluid typing); the CNS and blood on the clothing of the suspect are not shown to be the result of violence (no suggestion was given how CNS did end up on trousers and a shirt).

For these cases, the alternative scenarios follow three of the four general scenarios proposed in Section 1.1. These were: (1) ‘time of deposition’, (2) ‘another person performed the crime’ and (3) ‘innocent explanation’; ‘secondary transfer’ was not proposed. This makes sense, as in these cases, generous amounts of cellular material matching the victim(s) were found for which it is more difficult to invoke secondary transfer.

## 5. A Criminalistic View on Targets to Be Included in a Cell Typing Assay

Basically, there are two main reasons to include markers for a body fluid or an organ tissue in a cell typing assay: (1) the cell type can be questioned in forensic cases and (2) the cell type can be informative to an alternative, innocent explanation.

### 5.1. Cell Types Questioned in Forensic Cases

From the case descriptions in Section 4.1, it is evident that in sexual assault allegations, most often the presence of vaginal cellular material and menstrual secretions is questioned.

Semen (spermatozoa and/or seminal fluid) can be involved as well, generally in three circumstances: (1) as questioned body fluid in a sample in which semen is expected to be the predominant body fluid such as in a ejaculation stain on the body or clothing of the victim or inside a condom; (2) as a questioned body fluid in a sample containing a surplus amount of other cellular material such as in a vaginal, oral or rectal sample taken from the victim; or (3) as a background body fluid in a penile swab or finger/nail dirt sample that is examined for the presence of, for example, vaginal cellular material.

Under circumstance (1), semen will be the predominant cell type, and, in most cases, microscopic analysis will suffice to show presence of spermatozoa and generate a DNA profile for the semen donor. In circumstance (2), when a surplus amount of cellular material of the victim is present, differential extraction (DE), (in which a mild lysis to extract nucleic acids from non-spermatozoa cells, followed by a strong lysis to extract nucleic acids of the spermatozoa) is likely performed. Since the presence of semen is questioned, most often this DE method will suffice and produce a sperm fraction from which a DNA profile informative for the semen donor is derived. When confirmation of the presence of spermatozoa in the sperm fraction is needed, various methods can be applied such as microscopic analysis or MSRE analysis [154,155,210,211] on DNA extracted from the sperm fraction [212]

This is different in circumstance (3), where semen may be present in such amounts that it masks the presence of other body fluids. Here, it would be useful to apply a DE method that not only extracts DNA but also RNA and analyze the non-sperm RNA fraction for cell types other than spermatozoa (note that seminal fluid RNA will end up in this non-sperm RNA fraction as well). However, DE is hard to combine with RNA extraction as RNases are active during the mild lysis step unless an ample amount of strong RNase inhibitors is used [104].

Other body fluids of which the presence may be questioned, are saliva and peripheral blood. For blood, molecular analyses are mostly applied for discerning peripheral blood and menstrual secretion. Saliva may be assessed in allegations of licking, which means that often body locations are sampled (breasts, genitals, mouth), and mixed DNA profiles are expected.

To assess allegations of anal penetration, the identification of a rectal mucosa marker was described recently [213]. A rectal mucosa marker is to be preferred over markers for fecal matter as rectal mucosa provides a more direct link to invasive activities. Finding a marker specific for rectal mucosa is challenging since many body cavities and tubular organs in the human body are lined by mucous membranes (for instance, along the respiratory, digestive, and urogenital tract), an epithelial tissue that secretes mucus. Thus, specificity of mucous membrane genes may be an issue, they may prove general mucosa markers or cross-react with some other body fluid derived from a mucous membrane [98,214].

### 5.2. Cell Types Informing on an Alternative Scenario

The second reason to include markers for a specific body fluid in a cell typing assay is to inform an alternative, innocent explanation for the findings. Nasal secretion has a high cellular content and may therefore leave detectable amounts after secondary transfer, which may be proposed as an alternative scenario (H1: vaginal penetration by penis; H2: victim sneezes in hand, shakes hand of the suspect, suspect touches his penis during toilet visit). Thus, nasal secretion markers have been identified and included in body fluid typing assays [104,215]. In addition, the presence of nasal secretion and blood in a sample may indicate nosebleed blood, that can be a spontaneous occurrence not connected to a crime. Occasionally, nasal secretion may be among the questioned body fluid such as in case of expiration of blood (here saliva markers will also be informative) or when a nosebleed is caused in a fight.

Other body fluids for which markers have been identified are urine and sweat [128,216,217,218]. These two body fluids have a relatively low cellular amount and are likely of limited importance. Urine may contribute some cellular material in underpants; sweat can be deposited together with skin. Skin was one of the earlier cell types for which mRNA markers were identified [98,219,220,221,222], probably because contact traces are frequently submitted for forensic analyses. However, forensic practice in the Netherlands has shown that there is hardly any value in showing the presence of skin, as skin residues reside in almost all submitted samples. The presence of skin and DNA of a person of interest may be regarded mistakenly as an indication of direct contact, and due to the abundance of epithelial material in public and private items [42], secondary transfer is within the bound of possibility.

For the assessment of cases of violence, various tissue types can be included. CNS tissue (brain and spinal cord) is highly important to include in the assays because of the often-lethal consequences. Other organs such as liver, heart, kidney, lung, trachea, intestine, stomach, spleen can be included for chest and abdominal injuries, but blood, muscular tissue and/or adipose are informative for injuries anywhere on a body [223,224,225].

### 5.3. Fluids and Tissues Carry Multiple Cell Types

In a human body, there are ~210 cell types [226], and body fluids and organs contain several types of cells (semen may be an exception as semen carries besides spermatozoa only few other cell types).

As evidentiary stains may be minute, not all cell types may be represented in a sample, so it can be opportune to include markers for various cell types from one tissue or fluid in an assay. For example, CNS tissue comprises two major tissues: grey matter and white matter. Grey matter (~10% of the tissue in the brain volume) consists mainly of neuronal cell bodies (and unmyelinated axons and glial cells), while white matter (the deeper layer) mainly contains myelinated axons (and astrocytes and oligodendrocytes that produce myelin) [227]. Whether an evidentiary stain carries predominantly grey or white matter, will affect which markers can respond.

With body fluids, samplings may have a more even distribution of cell types, due to the liquid constitution. These have different biological functions that affect their localization. For example, red blood cells function within the blood (amongst others for carriage of oxygen) while white blood cells act mostly outside of the bloodstream (immune system). As a consequence, white blood cells crawl into the lymphatic system by a process denoted extravasation [228]). The lymphatic system is an extensive drainage network and several other forensically relevant body fluids may thereby carry white blood cells (e.g., nasal secretion [228]).

It is crucial to understand the biological function and performance on other body fluids or tissues for markers included in a cell typing assay.

## 6. Interpretation of Cell Typing Results

Generally, an evidentiary stain that is subjected for cell type analysis is also submitted for DNA profiling as one wishes to infer what cell type is donated by which person. This invokes a three-way interpretation: (1) the DNA profile; (2) the cell typing data and 3) the combination aiming to associate donor and cell type. The interpretation of DNA profiles has progressed tremendously through the application of probabilistic software [229,230,231,232,233] that use information on the number of contributors, allele frequencies, peak height, drop-in, dropout, degradation and more to provide a weight of evidence in the form of a likelihood ratio (LR). The LR includes a person of interest under H1 (H2 generally calculates the likelihood for an unknown, unrelated person). With the interpretation of cell typing data, several issues are to be considered: (1) limitations in sensitivity; (2) constraints for specificity; (3) degradation in evidentiary stains; (4) complications in case of a mixture of cell types and (5) providing an evidential value. These issues are discussed below, followed by developments on the association of donor and cell type.

### 6.1. Sensitivity, Specificity and Degradation Issues Affecting Interpretation

Ideally, a cell typing method has similar sensitivity for all cell types assessed in an assay, and a similar sensitivity as DNA profiling so that for all donors that give DNA information cell type information is derived as well. Over time, DNA profiling has become more and more sensitive and a full DNA profile can be generated from DNA amounts that equate to a few cells. The sensitivity of cell typing depends greatly on the type of marker that is used. DNA-based markers (those assessing methylation status) will at best be as sensitive as DNA profiling as both analyze features of the two genomic DNA copies. MSRE analysis [154,155,210,211] indeed approaches the sensitivity of DNA profiling, but methods that rely on a bisulfite conversion are much less sensitive as a large portion of DNA is lost during the conversion [146,147].

RNA and protein/ enzyme markers are expressed and translated from DNA and may be several folds more abundant than DNA although expression levels can vary highly for different genes, and abundance is further influenced by stability/ turnover rates. Environmental and physiological factors may cause variation between people and over time (an infection will increase the number of white blood cells and the mRNAs these cells contain). Thus, it appears inevitable that the sensitivity of markers targeting either the same or different body fluids will vary [121] despite careful marker selection and assay optimization. An extreme example is semen from an azoospermic male, which will provide RNA and proteins for seminal fluid but little DNA (or spermatozoa mRNAs/ proteins). Thorough validation by which understanding of marker performance is obtained is of pivotal importance.

Specificity issues can relate to human-specificity or tissue-specificity [234]. Methods that analyze nucleic acids, can most often be designed to be human (or at least primate)-specific, which is less feasible for tests based on enzyme activity or antibodies. Processes such as readthrough or spurious transcription giving rise to non-specific background expression of mRNAs may result in signals above the detection threshold for the assay, especially when samples are over-amplified (by using too much input or too many amplification cycles). Technological imperfections can also cause non-specific signals like remnants of DNA in RNA assays (this is only an issue when it is not possible to design cDNA-specific amplicons with for instance one primer spanning an exon-exon boundary) or incomplete bisulfite conversion in methylation-based assays. The most difficult form of non-specificity is when markers cross-react with cell types other than the intended cell type. This occurs more with body fluids than with organs. For instance, saliva, nasal secretion and vaginal material are produced in body cavities (oral, nasal, vaginal) that are all lined with mucous membranes. These can, as previously mentioned, give rise to expression of the same mucous markers in these body fluids (denoted general mucosa markers [98]). These non-specific responses can show inter-individual variation: mRNA vaginal markers were detected in nasal secretions in many donors, with no relation to gender or having a cold [104]. As with sensitivity, thorough validation with different donors is highly important to understand marker performance.

Outside the human body, factors such as high humidity, UV radiation and/or high temperature can invoke degradation of nucleic acids, especially when stains have not fully dried. DNA is more resistant to hydrolysis than RNA, as the 2’-OH group can attack the 3’-phosphate, leading to hydrolytic cleavage of the phosphodiester bond, which may lead to samples providing a DNA but no or hardly an RNA profile [97]. Amplicons in RNA assays are therefore generally shorter (preferably under ~150 base pairs) than in DNA typing (up to ~450 base pairs). Intramolecular base pairing within RNA molecules (not only G:C and A:U but also G:U) may result in secondary and tertiary structures that provide more stability to certain regions, and amplicons targeting such stable RNA regions (StaRs) [95,235] may enhance RNA profiling. DNA methylation assays have the advantage of using the same nucleic acid as used for DNA profiling, so there is no difference in stability (the methylation marks remain stable also in body fluids deposited outside a human body [236]).

### 6.2. Interpretation of Mixed Samples

RNA casework has shown that most evidentiary samples contain multiple fluids/ tissues, particularly with body fluid typing and less so with organ typing. One cause is that many samplings are taken from body locations (penis, fingers) or fabrics (clothing, bedding) that are likely to carry skin and other body fluid depositions. Moreover, the contributions may be highly unbalanced. To work on mixtures, markers should best respond in on-off modus meaning that a signal is obtained when the body fluid is present and not when a body fluid is absent. This can be achieved with RNA-based assays when markers are selected that show a large difference in expression between target and non-target tissues. Over-amplification needs to be avoided and detection thresholds need to be set.

For methylation-based assays, application to mixtures depends on the technology that is chosen. With MSRE, CpG targets are selected that are only methylated in the target tissue (this does not need to be a 100% methylation). PCR product can only be generated when the restriction enzyme is unable to cut the DNA due to methylation of the restriction site [237].

When methylation levels are assessed quantitively, for instance by sequencing bisulfite-converted DNA, it is highly complex to set interpretation guidelines for mixed samples. For example, when a chosen marker shows on average a 50% methylation rate in vaginal mucosa and 10% in other cell types, a 20% methylation level for the marker in an evidentiary sample can occur in different ways: (1) no vaginal mucosa is present and the methylation level of the other cell types (skin and/or semen) turns out higher than the average of 10%; (2) vaginal mucosa is present and no other cell types and the 20% methylation rate is an outlier for vaginal mucosa; (3) vaginal mucosa is mixed with other cell types for which a ratio of 1:3 would nicely explain the methylation rate of 20%. In this example, the DNA profile may be informative as a female donor will correspond to vaginal mucosa and the male donor to semen (if detected), but assumptions may need to made, for example, that no saliva from either one of the donors is present. When choosing a methodology for casework application, its suitability for mixed samples needs to be considered to enable appropriate interpretation.

### 6.3. Reporting Cell Typing Results

In expert reports, cell typing results are generally given as ‘indication for the presence of’ when genetic markers or tests for a body fluid or organ tissue are observed. With RNA typing, interpretation thresholds are applied as both drop-in and dropout may occur, due to limitations in sensitivity and specificity described above. These interpretation thresholds can include a numerical scoring [238], or a strategy of using replicates and a 50% rule in which at least half of the markers over all replicates need to be detected [111]. All have the drawback that they represent a ‘fall-of-the-cliff’ method, which means that the detection of one marker more or less may change the interpretation of the results.

Nowadays, the general trend in forensic reporting is to provide an evidential value, for example by presenting a likelihood ratio; the probability of the evidence given two competing hypotheses, H1 and H2. Formulating H1 can be straightforward (‘the sample contains vaginal cellular material’); formulating H2 is more complex as it could be ‘the sample does not contain vaginal cellular material’ or ‘the sample contains another body fluid’. These hypotheses follow the simplifying assumption of one body fluid per sample. More realistic hypotheses consider mixtures and could be formulated as H1: ‘the sample contains vaginal cellular material, and possibly other body fluids’ and H2: ‘the sample does not contain vaginal cellular material, but other body fluids’ (note that one could also specify these other body fluids). Conditioning on cellular material assumed to be present (for instance, because the sample is a penile swab) would generate hypotheses like H1: ‘the sample contains vaginal cellular material, and penile skin and possibly other body fluids’ and H2: ‘the sample does not contain vaginal cellular material, but penile skin and possibly other body fluids’.

Some statistical approaches have been proposed for body fluid typing; for organ typing, a multivariate statistical model was trained [120,239,240,241,242]. The basis of such models is an experimental dataset consisting of ground truth data, that should be casework representative. This is challenging as casework includes a wide range of samples for which the true composition is not known. It is not trivial to prepare such a dataset, and the LR should be calibrated to suit the size of the dataset (note that for DNA datasets the allele frequencies are used, while with RNA datasets an RNA profile is considered in its entirety). Reporting the LR in a verbal scale rather than a numerical value, seems more appropriate. Clearly, further development is needed, although the developments are promising.

### 6.4. Associating Donor and Body Fluid

A challenge for any forensic RNA typing method is the association of donor and body fluid. Complications are that one body fluid can be given by multiple donors and/or one donor can give multiple body fluids. Even with simple mixtures of two donors each giving one body fluid, it was shown that peak heights in DNA and RNA profiles cannot be used straightforwardly [97]. Only with gender-specific body fluids (vaginal cellular material and semen) and the involvement (or assumption) of one donor of that gender, is association of donor and body fluid possible. This introduces the risk of an association fallacy [2,243]. Thus, alternative procedures are being developed like SNPs (single nucleotide polymorphisms) in transcribed regions (RNA-SNPs) [242,244,245,246]. The concept is that the RNA indicates which cell type is present and that, as the RNA is transcribed from DNA, the genetic variation within this transcribed region provides information on the identity of the donor. First, which cell types are present is assessed, then the genotype for the RNA-SNPs of the donors that are assumed to be present are derived by genotyping their reference DNA (since this is DNA, there is no need to provide the body fluid questioned) and lastly the RNA of the stain is sequenced to see what SNPs are present in the transcribed mRNA to see if/ who of the donors matches this sequence.

This is a developing area with multiple challenges at hand: (1) SNPs can affect the stability or transcription level of RNAs, so the genotypes at the DNA and RNA level may not align (for instance one appears heterozygous at the DNA level but since the presence of a SNP affects RNA stability or expression level, one appears homozygous at the RNA level; (2) linked SNPs should be regarded according to their genetic phase (equivalent to a microhaplotype), which may be complicated when different SNPs reside in different amplicons; (3) the approach is only to be explored for samples in which all donors can be assumed as there is no clear strategy/consensus yet on how to deal with unknown donors. It is probably best to limit the approach initially to two-person mixtures.

CpG-SNPs (SNPs nearby CpGs indicative of cell types) have also been described [247,248]. Methods not depending on bisulfite conversion (such as MSRE) may be most practical to use to detect these as the bisulfite conversion will affect all non-methylated cytosines complicating SNP detection and primer design. A MS-HRM assay targeting DACT1 that is hypomethylated in semen was used [249] to determine if it was possible to associate semen with a DNA profile in a mixture. Some progress was made, and it was possible to determine whether the semen compromised the majority, almost half, or was in the minority of the components in a mixed fluid.

## 7. Further Considerations

### 7.1. Other Marker Types and Technologies That Have Been Explored

#### 7.1.1. Non-Coding RNAs

Various non-coding RNAs have been explored as markers for body fluid and tissue identification.

miRNAs are a class of small RNA molecules, 18–25 nucleotides in length, which are involved in the regulation (repression) of mRNA translation and stability [69,71]. with various forensic applications including body fluid identification [250].

Adult-specific roles for miRNAs (adult physiology, cancer development or suppression) have been described in various stem cell populations [251] and show continuous/ steady expression. This means these markers may be less specific for body fluid and tissue identification than might be suggested by the highly specific expression patterns seen in embryos [252]. Additionally, since human miRNAs generally interact through limited base pairing of only a few bases in the 5’ part of the miRNA, a miRNA can bind to many targets. Conversely, an mRNA may be targeted by several (different) miRNAs. For these reasons, a key limitation of miRNAs is their specificity. Nonetheless, many studies have proposed miRNAs for body fluids such as blood, saliva, menstrual blood and semen [253,254,255,256,257] and body tissues including brain, liver, skeletal muscle and skin [258]. miRNAs are stable [259] and have been shown to be detectable in forensic-like samples when treated to a range of environmental conditions [218,260] after laundering [261].

Methods to analyze miRNAs primarily include miRNA expression profiles to identify candidate markers, followed by qPCR to test specificity [253,255,256,258,262]. Some approaches include the extraction and analysis of miRNA and DNA together [263] and multiplexed miRNA panels [264] able to discriminate between venous blood, menstrual blood, semen, and saliva. High throughput sequencing of miRNAs from body fluid samples has also been used to identify new candidates [265]. Statistical methods and reference (endogenous) miRNA markers [262] are needed to measure miRNA expression with qPCR. Recently classification algorithms have been applied to the interpretation of miRNA profiles as detection is not a simple yes–no answer [259].

The longest known class of small non-coding RNA are the piwi-interacting RNA (piRNA), 26–31 nucleotides in length. Their role is in the transcriptional and post-transcriptional silencing of transposable elements which may be differentially expressed in different body fluids making them suitable candidates for identifying body fluids (although transposon silencing is foremost important in the germline where piRNAs were initially found [266]). Two papers [267,268] describe the characterization by RNA sequencing of piRNA expression profiles and the selection of three piRNA markers (piR-hsa-27622, piR-hsa-1207 and piR-hsa-27493) that distinguish between venous blood and menstrual blood and a further two (piR-hsa-27493 and piR-hsa-26591) to distinguish between saliva and vaginal material, implemented as TaqMan RT-qPCR assays.

Short interfering RNA (siRNA) are another class of small non-coding RNA with a role in gene expression [269]. To date there are no reports of their use as markers in forensic science, perhaps because their role is to interfere with the expression of specific genes, by degrading RNA after transcription, rather than to promote the expression or modification of specific transcripts.

Long non-coding RNAs (lncRNAs) are RNA transcripts not translated into proteins that appear to be involved in the regulation of gene expression. Approximately 78% of them are tissue-based compared to approximately 18% of mRNA transcripts. One of the best studied is the X-inactive-based transcript (XIST) involved in X-chromosome inactivation [270,271]. To date, XIST has been incorporated into an assay [272] to positively identify male and female cell material in forensic samples with a second study [273] further testing the assay on forensic type samples.

#### 7.1.2. Proteome

All body fluids and tissues contain unique proteomes, derived from the expression of mRNAs (see Section 3.1.1) in turn derived from the genome. In the past, unique proteins of each body fluid or tissue type have been detected and presumptively identified by determination of the enzyme activity (acid phosphatase for example) or immunological properties (RSID tests (Independent Forensics, Lombard, IL, USA) for example). More recently technological developments in proteomics, as for genomics, have highlighted potential benefits for forensic identification purposes and useful reviews of the forensic potential have been published [274,275,276].

Mass spectrometry (MS) is the analytical method of choice with advantages including high sensitivity and specificity [277]. Generally, candidate markers are found by *de novo* analysis or from reference proteomes, then validated by testing on known samples. By detecting multiple peptides in a single assay from more than one protein per fluid, the assay can be considered not only confirmatory but also human-based and suitable for mixtures. Sensitive and specific assays have been developed including blood, semen, seminal fluid, saliva, vaginal/ menstrual fluid and urine [277,278,279,280] using methods such as matrix-assisted laser desorption/ionization (MALDI) MS [277], quadrupole time-of-flight MS [278,279] MALDI MS Profiling and Imaging [281] and MALDI time-of-flight MS [282] from samples as diverse as blood-stained finger marks [281] and blood and vaginal cellular material under fingernails [282], although difficulties in identifying urine recovered from substrates and semen samples that had been mixed with lubricants were found [280]. A challenge to be addressed is that the protein content of each fluid/ tissue varies considerably, and whilst it is possible to detect very small amounts of blood in saliva, the opposite was not true [277,278,279].

MS methods tend to be destructive to the sample, however in a recent report [283] a new approach, sheath-flow probe electrospray ionisation MS was used, which allows stains to be sampled with no preparation or sample destruction bar the initial small amount taken. In the research which examined blood, saliva and urine, alterations were observed in the spectra over time suggesting that this approach could also be used to age a stain.

The relationship between the proteome and the genome offers the possibility for proteomic “genotyping” [284,285] in a similar manner to the suggested RNA SNP and CpG SNP approaches described in Section 6.4. This has clear advantages when DNA in a sample is compromised in quality and quantity such as in a hair shaft, as proteins are more stable and resistant to damage and decay than DNA. Genetic variations in human DNA can be linked to mutations found in hair proteins (genetically variant peptides (GVPs) referred to as single amino acid polymorphisms, SAPs, which in turn can be associated with their DNA counterparts (missense SNPs). GVPs containing SAPs can be identified by mapping these peptide sequences using MS and results have been obtained from short lengths of human hair [275,286]. Not only could this approach be used to “identify” and link individuals, but it can also be used to infer ancestry as SNP profiles varied between ancestral groups.

#### 7.1.3. Aptamers

Aptamers are single-stranded oligonucleotides or peptides that are selected using SELEX (systematic evolution of ligands by exponential enrichment) in a sequential process to bind their target molecules in a highly specific and sensitive manner. Initial studies used RNA aptamers [287,288], with the first description of a DNA aptamer in 1992 [289] and the first XNA aptamer capable of binding to a small molecule (ochratoxin A) in 2018 [290]. Aptamers have a number of advantages over assays such as the immunochromatogenic-based methods described in Section 2.2.1. Consistent aptamer synthesis methods, more straightforward functionalization, equivalent binding affinities and cheaper long term and stable production are all factors.

Aptamers are now widely used as biosensors in a large number of fields [291,292] with significant and recent examples seen for the rapid detection of SARS-CoV-2 [293,294] and have potential for the use in forensic science [295]. Aptamers have been developed to detect PSA [296], hemoglobin [297] and more recently, aptamers to detect spermatozoa have been described using a combination of Cell-SELEX and massively parallel sequencing technologies [295]. Once suitably specific and sensitive aptamers are obtained, they can be built into a range of sensors for detection many of them suitable for field deployment.

## 8. Concluding Remarks

Preferably, body fluid typing and DNA profiling (to answer the what and the who question) are analyzed on the exact same sample. This can be achieved when the DNA extract is used for cell type inference (as with methylation analysis) or when RNA is extracted from a fraction normally discarded during DNA extraction (like a flow through fraction when binding DNA to a silica column). Case reports have been published for both methylation [298] and RNA-based assays [299]. The advantage of using DNA for body fluid typing is that no additional extraction procedure is needed; the disadvantage is that less DNA remains for further analyses such as Y-STR profiling, mtDNA analysis or the prediction of age, ancestry, appearance. Microbiome analysis can use either DNA or RNA. The global COVID-19 pandemic has stimulated the development of assays that may find their use in forensic analysis such as multiplexed and extraction-free amplification assays [300], which may provide RNA-based tests prior to DNA extraction and analysis that may present the opportunity of triage. Such extraction-free methods [301] present pioneering opportunities beyond advancements such as one-step RT-PCR methods that simplify current methodology [302].

Introducing cell typing methodologies in a forensic laboratory requires extensive validation, implementation of interpretation and reporting, and explaining results for judiciary. This comes with additional costs (also because the technique itself may ask quite some hands-on laboratory work) and it has been suggested that it is best performed or outsourced to specialized laboratories.
